# Characterization, recellularization, and transplantation of rat decellularized testis scaffold with bone marrow-derived mesenchymal stem cells

**DOI:** 10.1186/s13287-018-1062-3

**Published:** 2018-11-21

**Authors:** Elias Kargar-Abarghouei, Zahra Vojdani, Ashraf Hassanpour, Sanaz Alaee, Tahereh Talaei-Khozani

**Affiliations:** 1 Tissue Engineering Lab, Department of Anatomical Sciences, School of Medicine, Shiraz University of Medical Sciences, Zand St., Shiraz, Fars 7134845794 Iran; 2 Laboratory for Stem Cell Research, Department of Anatomical Sciences, School of Medicine, Shiraz University of Medical Sciences, Shiraz, Iran; 3 Reproductive Biology Department, School of Advance Sciences and Technology, Shiraz University of Medical Sciences, Shiraz, Iran

**Keywords:** Testis, Decellularization, Scaffold, Mesenchymal stem cell

## Abstract

**Background:**

Regenerative medicine potentially offers the opportunity for curing male infertility. Native extracellular matrix (ECM) creates a reconstruction platform to replace the organs. In this study, we aimed to evaluate the efficiency of the testis decellularized scaffold as a proper niche for stem cell differentiation toward testis-specific cell lineages.

**Methods:**

Rats’ testes were decellularized by freeze-thaw cycle followed by immersion in deionized distilled water for 2 h, perfused with 1% Triton X-100 through ductus deferens for 4 h, 1% SDS for 48 h and 1% DNase for 2 h. The decellularized samples were prepared for further in vitro and in vivo analyses.

**Result:**

Histochemical and immunohistochemistry studies revealed that ECM components such as Glycosaminoglycans (GAGs), neutral carbohydrate, elastic fibers, collagen I & IV, laminin, and fibronectin were well preserved, and the cells were completely removed after decellularization. Scanning electron microscopy (SEM) showed that 3D ultrastructure of the testis remained intact. In vivo and in vitro studies point out that decellularized scaffold was non-toxic and performed a good platform for cell division. In vivo implant of the scaffolds with or without mesenchymal stem cells (MSCs) showed that appropriate positions for transplantation were the mesentery and liver and the scaffolds could induce donor-loaded MSCs or host migrating cells to differentiate to the cells with phenotype of the sertoli- and leydig-like cells. The scaffolds also provide a good niche for migrating DAZL-positive cells; however, they could not differentiate into post meiotic-cell lineages.

**Conclusion:**

The decellularized testis can be considered as a promising vehicle to support cell transplantation and may provide an appropriate niche for testicular cell differentiation.

## Background

Reproductive disorders are common in population, and they fuel many socioeconomic problems. Nowadays, the couples with male factor infertility are also increasing by changes in the lifestyle and exposure to different endocrine interrupters. Assisted reproductive techniques (ART) offer the opportunity to cure many reproductive disorders, but in many cases, the fertility rescue fails to happen; therefore, more effective procedures are needed. Stem cell therapy [1] and tissue engineering [2] are two new approaches to improve the male fertility. Regenerative medicine and tissue engineering offer the opportunity to culture various cells into the appropriate biomaterials and recapitulate the naïve microenvironment for normal cell and tissue differentiation and function. Based on the type of disorder, 25–75% of the cases such as testicular sperm extraction can be treated by ART [3]. However, more severe forms of infertility such as spermatogenesis arrest [3] need more sophisticated attention. Sperm derivation from autologous stem cells can be a potential treatment of such cases. Finally, perhaps 1 day, the treatment of infertility can be individualized.

Recapitulation of the testis microenvironment for spermatogenesis was the subject of some investigations. For instance, the proliferation and self-renewal of the spermatogonial stem cells were improved by culturing in 3D agarose hydrogel [4]. Isolated cells from the rat testis have been reported to be reconstructed by culturing in a three-layer gradient matrigel [5]. Also, poly-l-lactic acid nanofibers were reported to be used as basement membrane to improve the frozen-thawed mouse spermatogonial stem cell viability [6]. The germ cells have been detected to differentiate into late spermatid within a 3D collagen and agar culture system [7]. Besides, 3D collagen/alginate hydrogel improved the embryonic stem cell-derived primordial germ cell differentiation [8]. 3D culture systems can be considered as a biomimicry of the testis microenvironment and improve the spermatogonial stem cell differentiation condition. The extracellular matrix and basement membrane constitute the main niche for germ cell as well as sertoli and interstitial cell that support their functions [9]. A culture system contained laminin and fibronectin, but not collagen along with meiosis inducer, and sertoli cell co-culture led to an elevation in the spermatogonia stem cell marker expression such as DAZL by chick embryonic stem cells [10]. Glycosaminoglycans (GAG) such as heparin sulfate facilitated the primordial germ cell migration and survival [11]. Acellular matrix is usually prepared by removing the cellular components from the tissue through mechanical and/or chemical methods [12, 13]. The testis has been previously shown to be decellularized successfully with good extracellular matrix (ECM) preservation [14]. Decellularized tissues with naïve microarchitecture can be considered as an appropriate niche for germ cell differentiation and has been reported to be used for adult testicular tissue culture [2]. The decellularized tissues degrade slowly after implantation and are generally replaced by the host cell-derived ECM [15].

In patients at the final stage of organ failure, biopsy of the tissue cannot adequately provide the necessary cell for transplantation [15]. The decellularized scaffolds can be recellularized by either culturing the isolated cells in vitro or cell migration from the host tissue in vivo. Different stem cells can be substituted as a source for germ cell differentiation, especially for those who suffer from spermatogonia disorders.

Among adult stem cells, mesenchymal stem cells (MSC) have been mostly studied. They can be easily extracted from the bone marrow, and the most important characteristic of these cells is their ability to maintain homeostasis and integrity of the tissues [16]. The stem cells are located in specific niche, where they would be able to make normal cell replacement, as well. If a lesion occurs in the tissue, they will have the capacity to migrate to the site and differentiate to tissue-specific cells or produce cytokines that repair the tissue [17]. The role of mesenchymal stem cells in the immunomodulation has already been recognized [18]. Therefore, if the tissue scaffold is recellularized with the MSCs, they will escape from the host’s immune system [19, 20].

In this study, we decellularized and characterized the rat testis, and for the first time, we transplanted the recellularized scaffold with bone marrow-derived MSCs to investigate their possible capability to induce differentiation of the testis-specific cells including the germ cell, sertoli, leydig, and post-mitotic spermatogenesis lineage.

## Materials and methods

### Decellularization of rat testis

The testis samples were collected from Sprague-Dawley rats weighing 150–200 g. Thereafter, the testes were washed with phosphate-buffered saline (PBS) three times and frozen at − 80 °C.

After thawing, the samples were immersed in deionized distilled water (DDW) for 2 h and perfused with 1% Triton X-100 (Sigma, USA) in DDW through ductus deferens for 4 h followed by 1% SDS (Sigma, USA) in DDW for 24 h at room temperature, using peristaltic pump (Heidolph, Germany) at a rate of 0.5 cc/min. Then, the perfusion rate was elevated to 0.9 cc/min for another 24 h. To minimize the remnant of residual DNA, 1% DNase (Sigma) in PBS (pH 7.2) was perfused with the same aforementioned condition for 2 h. The treated samples were washed in DDW for an additional 4 h and then prepared for further analyses. For in vitro and in vivo studies, the samples were sterilized with 3% peracetic acid in 4% ethanol, washed with PBS, and finally lyophilized by freeze–drier (Christ Alpha 2-4 LD-plus, Osterode am Harz, Germany) at − 50 °C.

### Decellularization efficiency

The paraffin-embedded sections were prepared from decellularized and intact testes to evaluate the efficiency of decellularization procedure and ECM preservation. The sections at 5 μm thickness were stained with Hoechst (Sigma–Aldrich) and hematoxylin and eosin (H&E) to assess the devoid of cellular components, azan and aldehyde fuchsin staining to assess the collagen and elastic fiber preservation.

### Retention of carbohydrates

In order to identify the retention of acidic GAGs and neutral carbohydrates, we stained the decellularized tissues with 1%alcian blue (Sigma–Aldrich) at pH 1 and periodic acid–Schiff, respectively. The sections stained with alcian blue were, then, counterstained with nuclear fast red (Sigma–Aldrich).

### Immunohistochemistry

To evaluate the retention of collagen type I and IV, laminin and fibronectin, the decellularized and intact testes were fixed in 4% paraformaldehyde for 24 h followed by immersing in 30% sucrose as a cryoprotectant for 72 h; at the end, they were transported to the liquid nitrogen. The tissues were then embedded in OCT and sectioned at a thickness of 6–7 μm. Endogenous peroxidase was neutralized by 0.3% H_2_O_2_ in methanol, and non-specific binding sites were blocked by 4% goat serum in PBS. The samples were incubated with anti-collagen IV (pre-diluted; ab6581, Abcam), anti-collagen I (pre-diluted; ab6577, Abcam), anti-laminin (pre-diluted; ab6571, Abcam), and anti-fibronectin (pre-diluted; ab6584, Abcam) antibodies overnight at 4 °C. Then, they were washed with PBS three times for 15 min. Finally, the samples were treated with streptavidin–horseradish peroxidase complex followed by incubation with diaminobenzidine (DAB, Dako) and counterstained with hematoxylin.

### DNA content analysis

To investigate the DNA content of the intact and decellularized testes (*n* = 3), dsDNA Assay Kit (QIAGEN, Germany) was used according to the manufacturer’s guideline. Briefly, the dried samples were cut into pieces with less than 25 mg in weight, transferred into the strips, and digested with proteinase K at 56 °C. After washing, the samples were added to 200 μL of 96% ethanol and DNA extracted by DNeasy Mini spin column. After DNA elusion, the DNA/protein ratio was evaluated by a spectrophotometer (Nanodrop Technologies Inc., Wilmington, USA) at 260/280 nm.

### Scanning electron microscopy

In order to evaluate the microarchitecture of the decellularized scaffolds, scanning electron microscopy (SEM) was performed. The samples were fixed in 2.5% glutaraldehyde for 24 h at 4 °C, dehydrated in increasingly graded ethanol, and dried with increasingly graded hexamethyldisilazane. Finally, the samples were covered with gold using Q150R- ES sputter coater (Quorum Technologies, UK) and imaged by a VEGA3 microscope (TESCAN, Czech Republic).

### Mesenchymal stem cell isolation

Mesenchymal stem cells were collected from the bone marrow of the adult rat. The rats were euthanized by chloroform, and the femur and tibia were removed. In order to collect MSCs, we inserted a 23-gauge syringe into the bone cavity and flashed with serum-free Dulbecco's modified Eagle's medium (DMEM, GIBCO). After centrifuging at 1200 rpm for 10 min, the bone marrow cells were re-suspended in 1 mL DMEM that was supplemented with 10% fetal bovine serum (FBS), 10 U/mL penicillin, 10 μg/mL streptomycin, and 1% l-glutamate (all supplied by GIBCO). Finally, the number of viable cells was checked with trypan blue and hemocytometer and transferred to culture dishes. The suspended cells were removed after 24 h, and the attached cells were allowed to grow up to confluency.

### Cytotoxicity assay

The sterile lyophilized decellularized testes were cut transversely into 5-mm pieces and re-sterilized by UV. At the third passage, bone marrow-derived MSCs were harvested and loaded at a density of 5 × 10^4^ per each scaffold for 24, 48, and 72 h. The same number of MSCs was seeded in a 2D conventional condition as the control culture. The cells in both 2D conventional and 3D conditions cultured DMEM supplemented with 10% FBS, 10 U/mL penicillin, 10 μg/mL streptomycin, and 1% l-glutamate. After incubation, the viability of the cells on the decellularized testis was determined by adding 1 mg/mL 3-(4, 5-dimethylthiazolyl-2)-2, 5-diphenyltetrazolium bromide (MTT, M5655; Sigma–Aldrich) for 3 h. Formazan was eluted by adding dimethyl sulfoxide (Sigma–Aldrich) for 15 min. The optical density of the eluted MTT was evaluated at 590 nm.

### Recellularization and in vitro assessments

To prepare the lyophilized decellularized scaffolds, we pre-conditioned them with DMEM supplemented with charcoal-stripped FBS overnight at 37 °C and 5% CO_2_. MSCs at a density of 5 × 10^7^/mL in DMEM supplemented with 10% FBS, 10 U/mL penicillin, 10 μg/mL streptomycin, and 1% l-glutamate were loaded on each scaffold. After 30 min, DMEM was added to the cell-seeded scaffolds and incubated at 37 °C and 5% CO_2_ for 3, 7, and 14 days. In order to avoid the effect of FBS steroid hormones on the MSC differentiation fate, the FBS was stripped by activated charcoal and dextran (Sigma) for 2 h.

The recellularized scaffolds were prepared histologically and stained with H&E and immunostained with anti-DAZL (1:100; Abcam; ab34139) antibody followed by incubating with Alexa-Fluor®488 conjugated secondary antibody (1:500; Abcam; ab150077). Then, the samples were counterstained with Hoechst. The intact testis was used as positive control.

### In vivo assessments

For in vivo transplantation, the cells were trypsinized at passage 3 and labeled with PKH26 red fluorescent cell linker kit (Sigma). The labeled MSCs at a density of 5 × 10^7^/mL were loaded into the pre-conditioned scaffolds. After 48 h, the scaffolds with or without cell loading were transplanted into different positions including the skin, renal capsule, mesentery, and liver. The rats were anesthetized by intramuscular injection of 10 mg/kg xylazine and 100 mg/kg ketamine; an incision was made in the abdominal wall and the recellularized scaffolds with or without cells was transplanted. The scaffolds were sutured using 8-0 nylon sutures, and the abdomen was closed using 3-0 nylon sutures. They also received analgesic drug and antibiotic. The animals were kept under the standard condition and ad libitum for 20, 40, and 60 days (*n* = 5 in each group). Then, all animals were sacrificed and the transplanted tissues were removed and prepared histologically. The sections were stained with H&E, and they were also immunostained for DAZL, inhibin, and CD68. The DAZL was performed in the same way as we did for in vitro samples. The inhibin and CD68 (ready to use, Dako) were detected by avidin–biotin–peroxidase complex method. Diaminobenzidine (Master Diagnostic, Spain) was used as chromogen, and the samples were counterstained with hematoxylin. The area that was occupied by seminiferous tubules and also the percentages of the fibrous seminiferous tubules were estimated by Image J software (http://mac.softpedia.com/get/Graphics/ImageJ.shtml). The possibility of post-mitotic cell differentiation was evaluated by interaction of the potential developing acrosome with peanut agglutinin (PNA, Sigma) lectin. The deparaffinized sections were incubated with 10 μg/mL of FITC-conjugated PNA for 2 h at room temperature, and then, they were counterstained with Hoechst.

To detect the endothelial cells and angiogenesis, we also incubated the slides with the 10 μg/mL of FITC-conjugated UEA lectin for 2 h at room temperature followed by counterstaining with Hoechst.

### Statistical analyses

The data were presented as mean values ± standard deviation, and statistical analyses were performed using one-way ANOVA analysis, LSD, and Mann–Whitney *U* test. GraphPad Prism was used for analyses. A *p* value less than 0.05 was considered as significant difference.

## Results

### Evaluation of decellularization

**Fig. 1:**
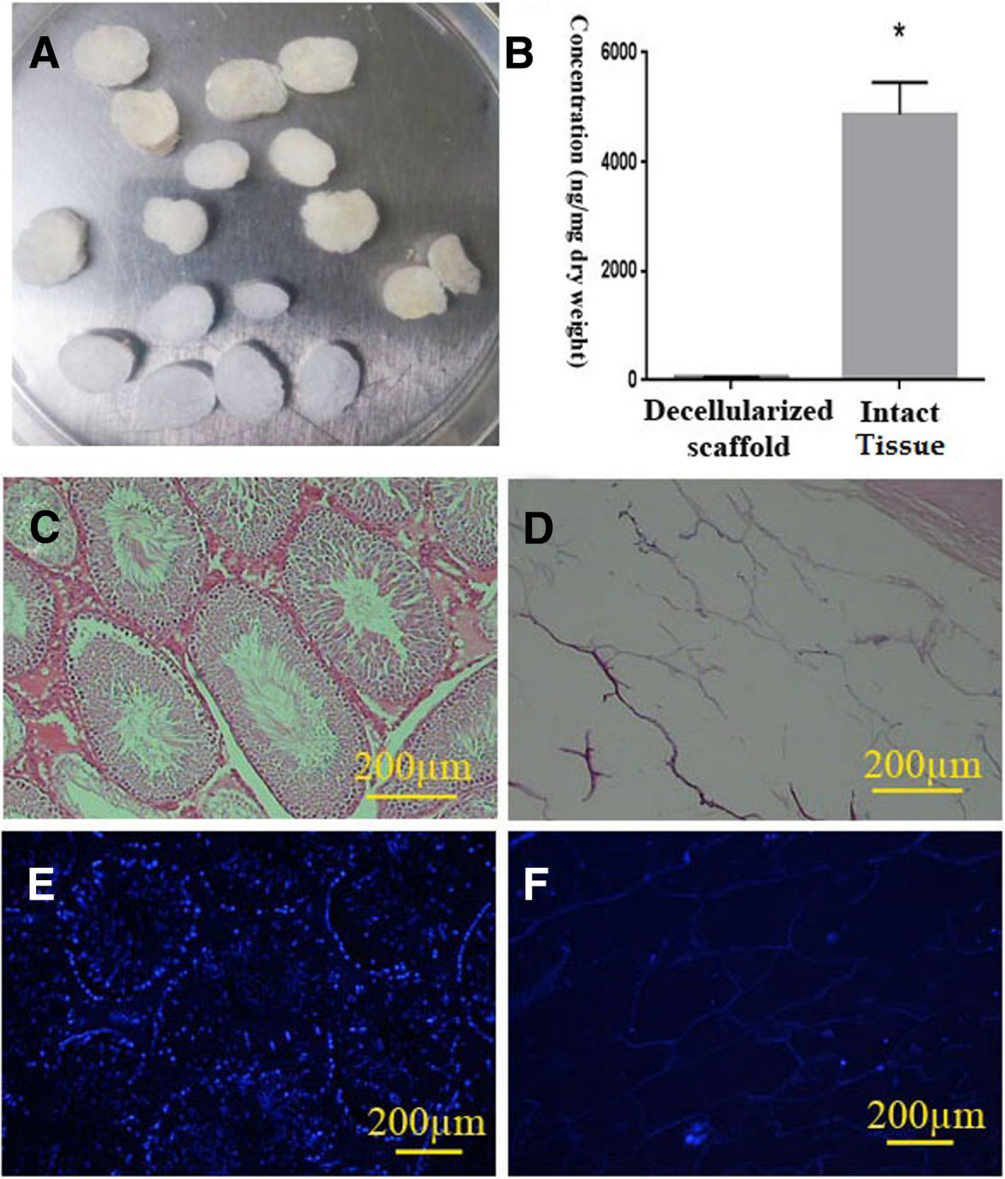
Macroscopic and microscopic structure of the rat testis after SDS-based decellularization process. A lyophilized decellularized testis scaffold showed whitish translucent appearance (**a**). The transverse section of the decellularized testis showed intact ECM and tunica albuginea gross architecture. DNA quantification showed significant cell removal by decellularization procedure (*N* = 3) (**b**). Comparing of the H&E (**c**, **d**), Hoechst (**e**, **f**), staining of the naïve (**c**, **e**), and decellularized tissue (**d**, **f**) also confirmed the cell removal. Both staining showed that ECM was preserved with no residual nuclei or cells

The first step in the assessment of the decellularization efficacy is to confirm the elimination of the cellular elements. Gross examination of the decellularized scaffold revealed that 1% SDS turned the color of the testis to the whitish translucent appearance (Fig. 1a). Quantification test also suggested a significant decrease in the DNA content of the decellularized testis (less than 50 ± 12 ng/mg dry weight) compared with that in the native tissue (Fig. 1b). H&E staining confirmed the cell removal along with the partially intact ECM architecture (Fig. 1c, d). Hoechst staining also showed the presence of a very few nuclei in each section (Fig. 1e, f). The tunica albuginea remained intact; however, the seminiferous tubules and interstitial tissue were detected with mild distortion. Vasculature framework without endothelial cells also preserved in the samples.

**Fig. 2:**
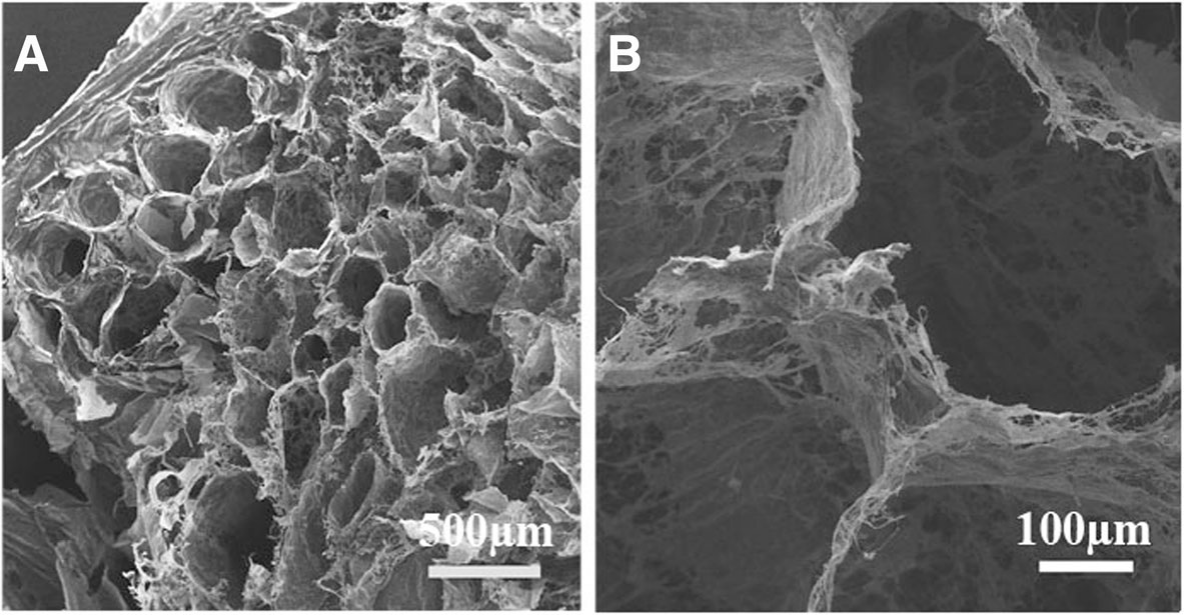
Scanning electron microscopy evaluates three-dimensional testis structure after decellularization. Ultrastructure of the decellularized testis confirmed that the framework of the testis remained intact and the testis tubules are visible on the transverse section (**a**). Higher magnification of a seminiferous tubules are shown without any cell, and the collagen fiber orientation and structure are almost unaffected (**b**)

Scanning electron microscopy was used to qualitatively evaluate the three-dimensional structure of the decellularized testis. Ultrastructure of the decellularized testis showed the convoluted, loop-shaped, tubular seminiferous tubules. Compact collagenous structure of the tunica albuginea remained nearly intact after the decellularization process (Fig. 2a). The SEM images confirmed the intact testicular three-dimensional ECM architecture and normal interstitial tissue without any cell or nucleus component (Fig. 2a, b).

### Characterization of the ECM preservation

**Fig. 3:**
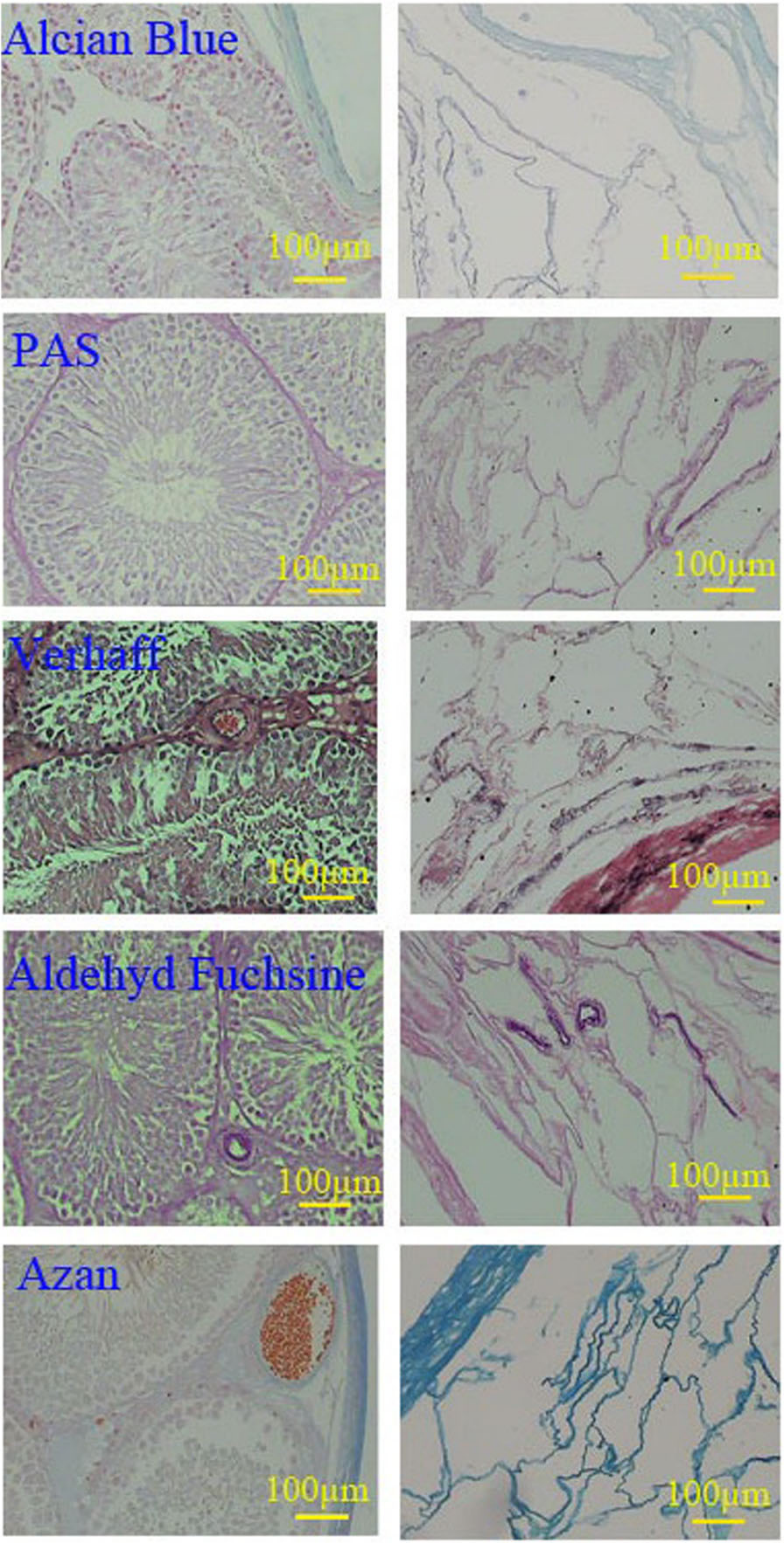
Comparison of the ECM content of intact testis (left series) with decellularized scaffolds (right series). Alcian blue and PAS showed that GAG and neutral carbohydrate were preserved after decellularization. GAGs were mainly present in tunica propria as well as tunica albuginea. Neutral carbohydrates were mainly present in the interstitium. Aldehyde fuchsin, Verhoeff, and azan confirmed the preservation of the collagen and elastic fibers as well. Collagen fibers were distributed in both tunica albuginea and tunica propria, but the elastic fibers were present in the wall of arteries and interstitium

Histochemical studies indicated that the ECM constituents were preserved properly in the decellularized testis. Alcian blue staining revealed that GAG distribution in decellularized testis was the same as that in the native one. GAGs were preserved in the wall of the vessels as well as in the tunica albuginea after decellularization. PAS staining showed that neutral carbohydrates were present in the interstitium and in the wall of the vessels of intact testis and they were preserved properly in the same position in the decellularized testis. Elastic fibers were distributed in the interstitium, the wall of the arteries, and tunica albuginea of native testis. Both Verhoeff and aldehyde fuchsin staining confirmed the preservation of the elastic fibers in the same regions of the decellularized testis (Fig. 3).

**Fig. 4:**
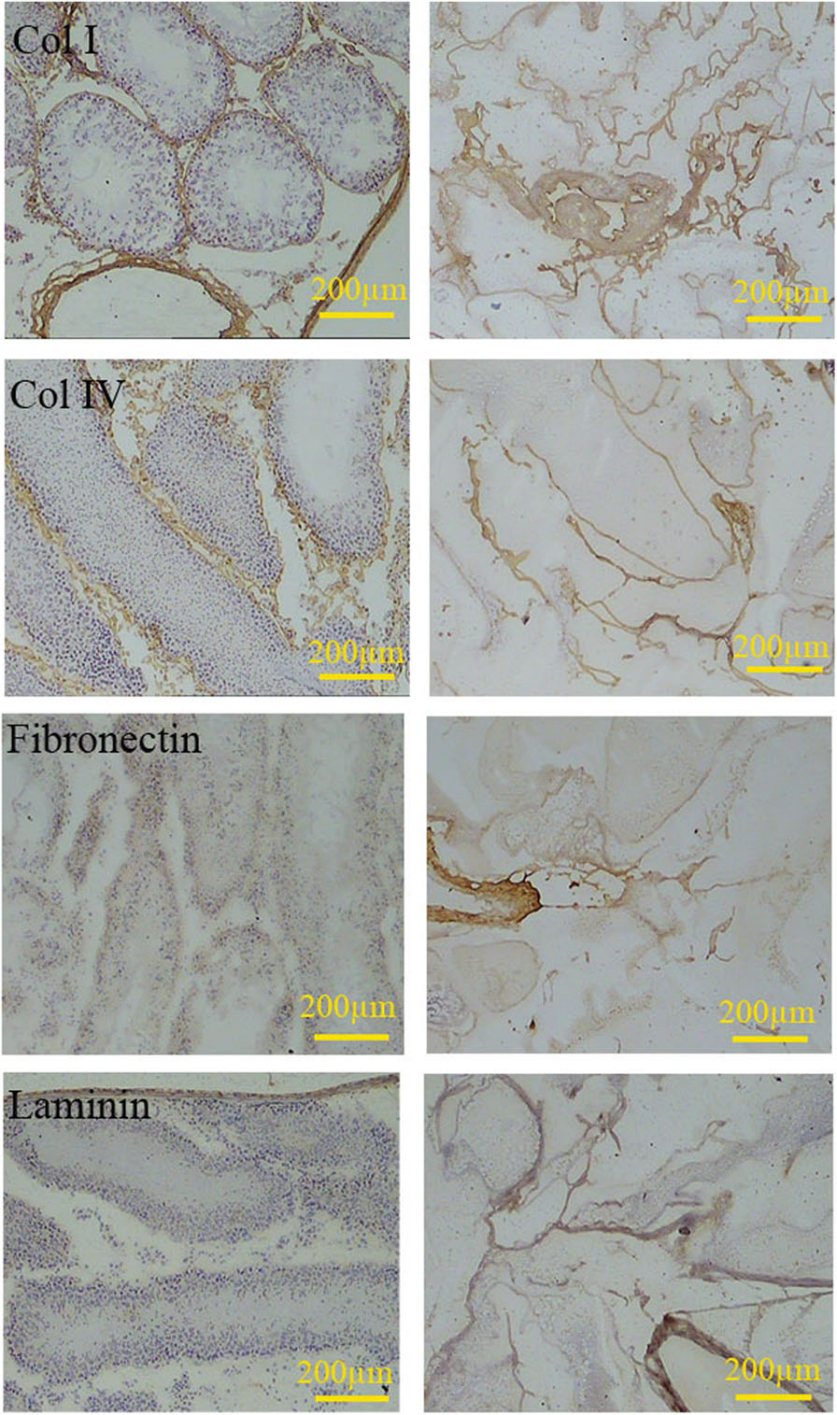
Comparison of the ECM content of the intact testis (left series) with decellularized scaffolds (right series) showed that collagen I, collagen IV, fibronectin, and laminin remained after decellularization. The distribution of these proteins was the same in both cellularized and decellularized tissues. The laminin and collagen IV were mainly present in the basal lamina, while the fibronectin distributes in all regions uniformly

Both azan and immunostaining revealed that collage type I was also preserved in the tunica propria, interstitium, the wall of the vessels, and tunica albuginea (Figs. 3 and 4). Immunostaining also indicated that laminin and collagen type IV were present in the basal lamina of the seminiferous tubules in both decellularized and intact testis. In both native and decellularized tissue, collagen type IV and fibronectin were present in the interstitium. Both fibronectin and laminin were preserved in the tunica albuginea and the wall of the large blood vessels in the decellularized tissue (Fig. 4).

### Cytocompatibility

**Fig. 5:**
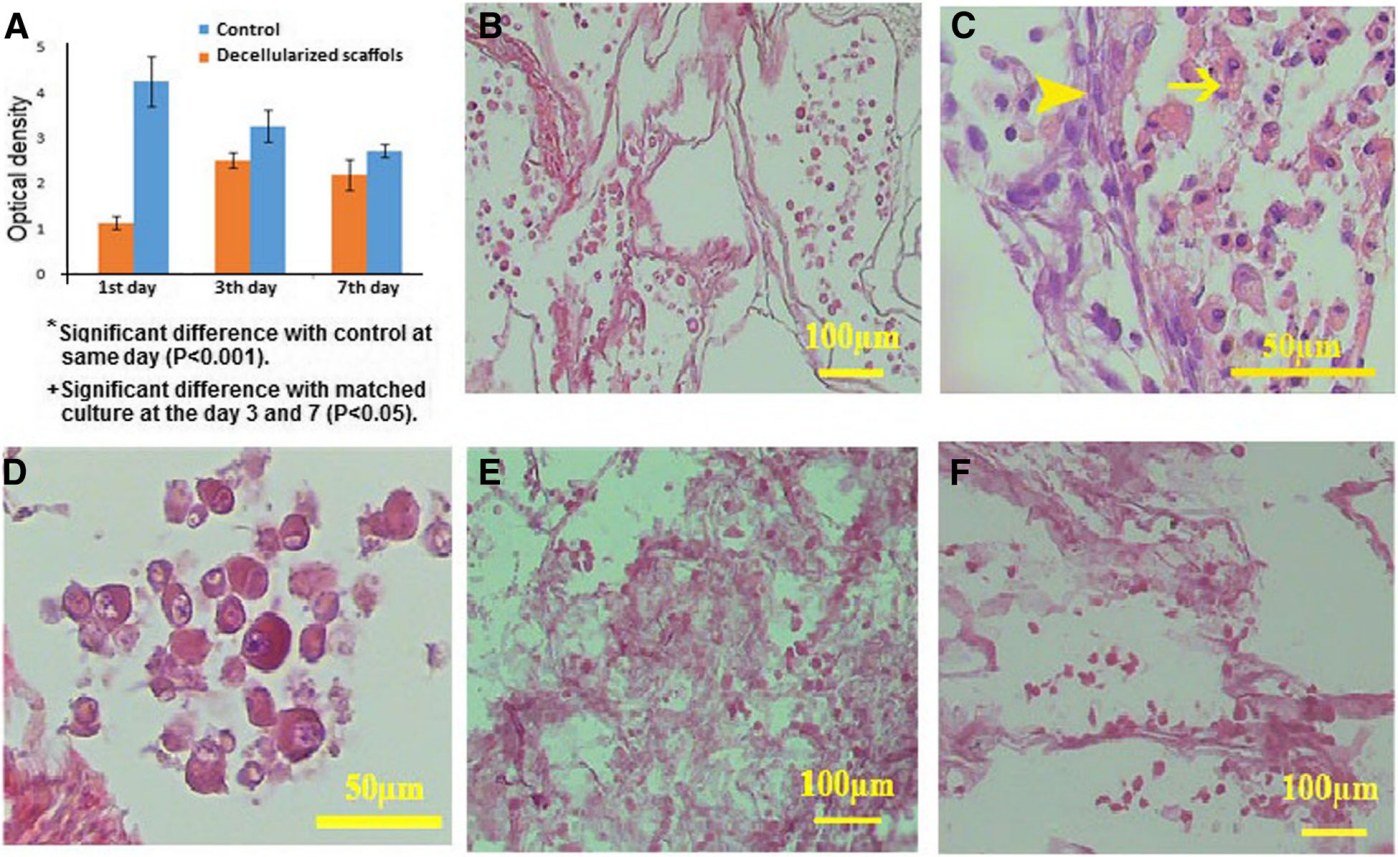
In vitro culture of recellularized scaffolds. MTT assay of the decellularized scaffold revealed that the scaffolds were non-toxic. At the day 1, the viability was significantly lower in the cells seeded on the scaffold compared to those seeded in 2D conventional monolayer cultures. At the days 3 and 7, the proliferation and viability significantly declined in 2D condition, while it significantly elevated in 3D condition. The similar cell viability was recorded in 3D and 2D culture conditions after 3 and 7 days (**a**). After 3 days, the cells within the scaffold showed various phenotypes according to the position (**b**); those located within the seminiferous tubules were spherical with round nuclei and acidophilic cytoplasm (arrow head), while those located in the interstitium were fibroblast-like (arrow, **c**). The higher magnification of the seminiferous tubules showed the spherical cells (**d**). After 7 (**e**) and 14 (**f**) days, the cells were present within the scaffolds; however, the cell density declined

MTT assay revealed that the decellularized scaffold was non-toxic. Bone marrow-derived MSCs were seeded into the decellularized testis and the cell viability was compared with that in 2D monolayer condition after 1, 3, and 7 days. In short term, the viability was significantly lower in the cells seeded on the scaffold compared to those seeded in 2D conventional monolayer cultures (*P* < 0.0001); however, in long term, it was the same in both 3D and 2D conditions. As the time passed, the proliferation and viability significantly declined in 2D condition (*P* = 0.0062 for the 1st day versus the 3rd day, *P* = 0.0006 for the 1st day versus the 7th day), while it significantly elevated in 3D condition (*P* < 0.0001 for the 1st day versus the 3rd day, *P* = 0.0244 for the 1st day versus the 7th day). The comparison of the cell viability in 2D and 3D conditions showed they were similar on days 3 and 7 of incubation (Fig. 5a).

### In vitro study

After day 3, the cells in the recellularized scaffolds were distributed with different phenotypes in both seminiferous tubules and interstitium (Fig. 5b). The cells located in the interstitium had a fibroblast-like structure with oval pale nuclei (Fig. 5c). The cells scattered within the seminiferous tubules were spherical with round or oval nuclei and acidophilic cytoplasm (Fig. 5c, d). Therefore, it seems that the ECM architecture and content regulated the cell morphology. In spite of cell morphology, the cell density decreased after 7 and 14 days (Fig. 5e, f). Besides, the cells that attached to the surface of the tunica albuginea were squamous in shape. Some mitotic figures were also observed in the cells loaded in the seminiferous tubules.

**Fig. 6:**
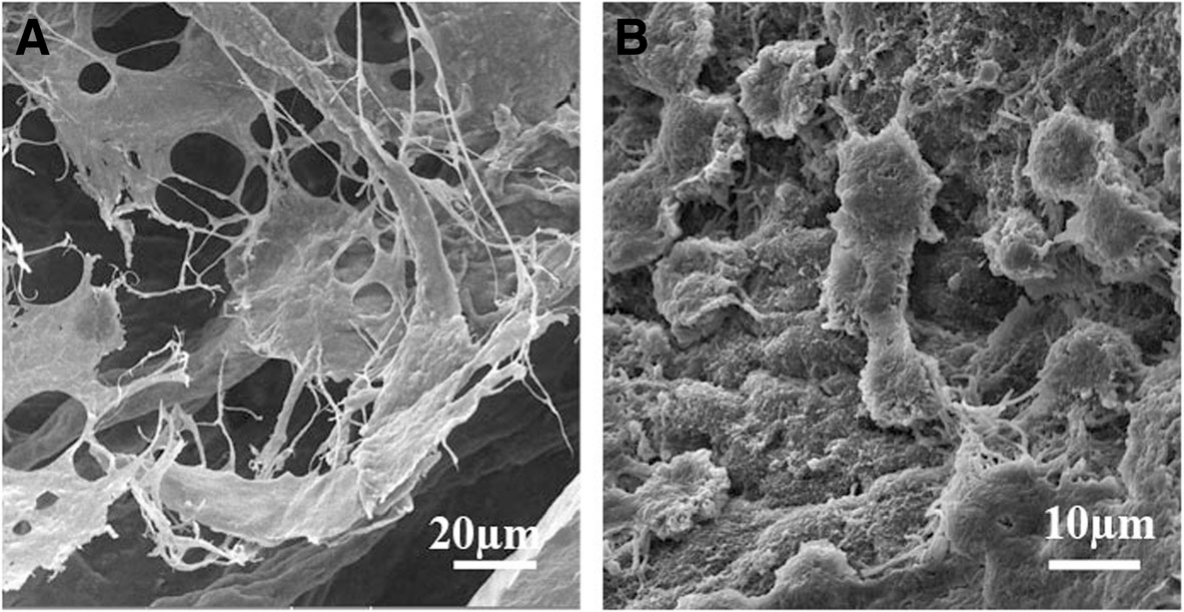
Scanning electron microscopy of the different regions of the recellularized scaffolds. Electron micrographs revealed that the cells were attached to the scaffolds; the cells located within the interstitium showed a cell body with numerous processes (**a**), while those located within the seminiferous tubules were spherical (**b**)

SEM images also confirmed the difference in the cell phenotype according to the location within the scaffolds. While the cells located within the interstitium showed a cell body with numerous processes (Fig. 6a), those located within the seminiferous tubules were spherical (Fig. 6b). Immunostaining showed that both seeded cells within the seminiferous tubules and interstitium of the scaffolds were DAZL-negative in all days.

### In vivo study

**Fig. 7:**
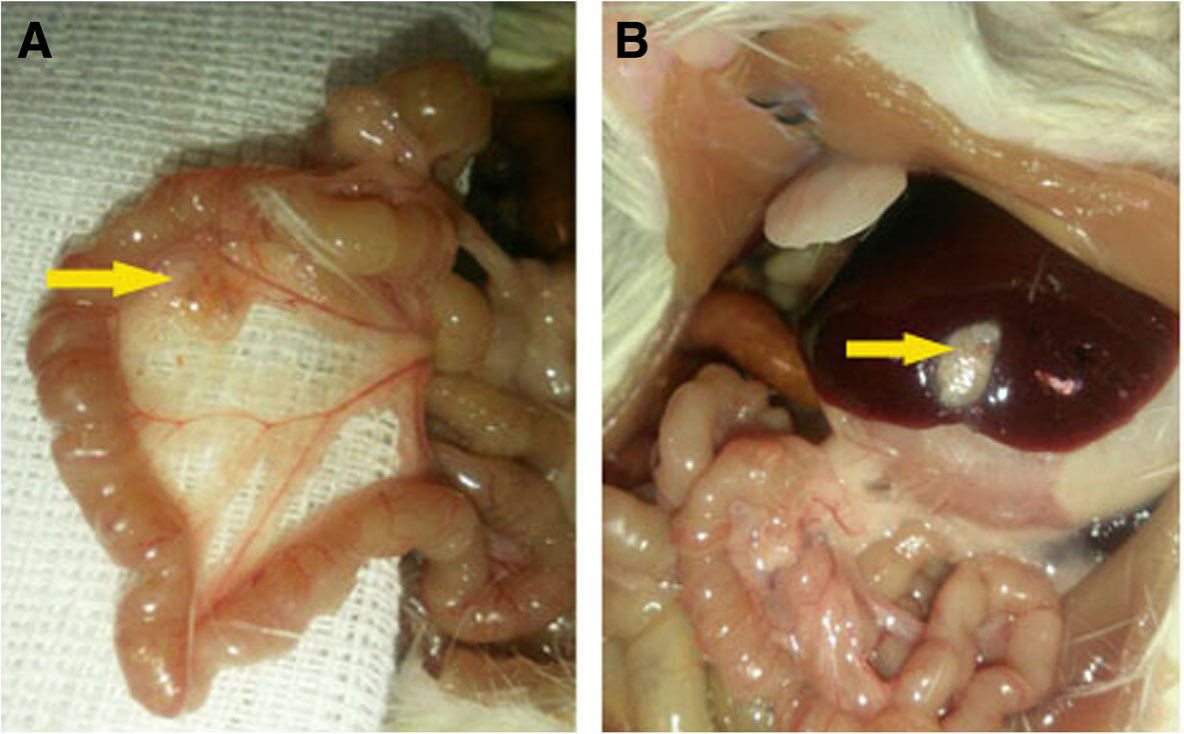
The scaffolds (arrows) transplanted on the mesentery (**a**) and liver (**b**) after 20 days. No obvious sign of gross inflammation or adhesion was observed

Gross examination of the grafts showed that there was no sign of inflammation or adhesion in those implanted in the liver; however, in some cases, the implant in the mesentery led to adhesion (Fig. 7). Besides, the cell-free and cell-seeded scaffolds were absorbed totally and replaced by connective tissue when they were implanted in renal subcapsular and subcutaneous regions without any sign of inflammation. Those implanted within the mesentery or liver remained up to day 60.

**Fig. 8:**
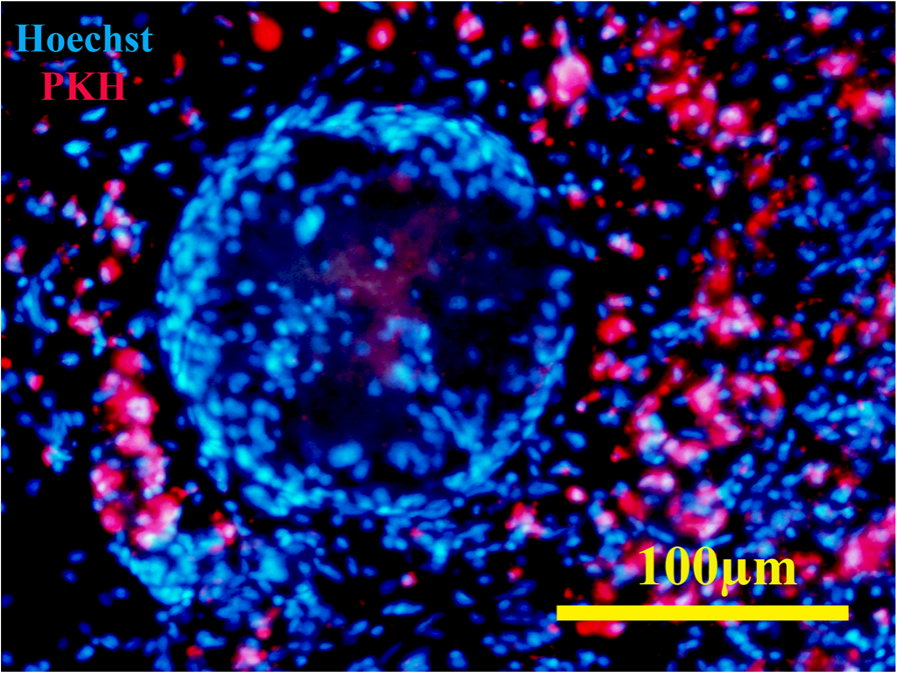
Cell tracing by PKH staining showed that the donor cells remained viable in the transplanted scaffolds. It was also showed that both bone marrow-derived MSCs (red) and host migrating cells were present in the implanted scaffold

The cell tracing by PKH showed that both bone marrow-derived MSCs and host migrating cells were present in the implanted scaffolds after day 20. Although most of the cells were present in the seminiferous tubules migrated from the host, both migrating and labeled MSCs could be found in the interstitium of the scaffolds (Fig. 8).

**Fig. 9:**
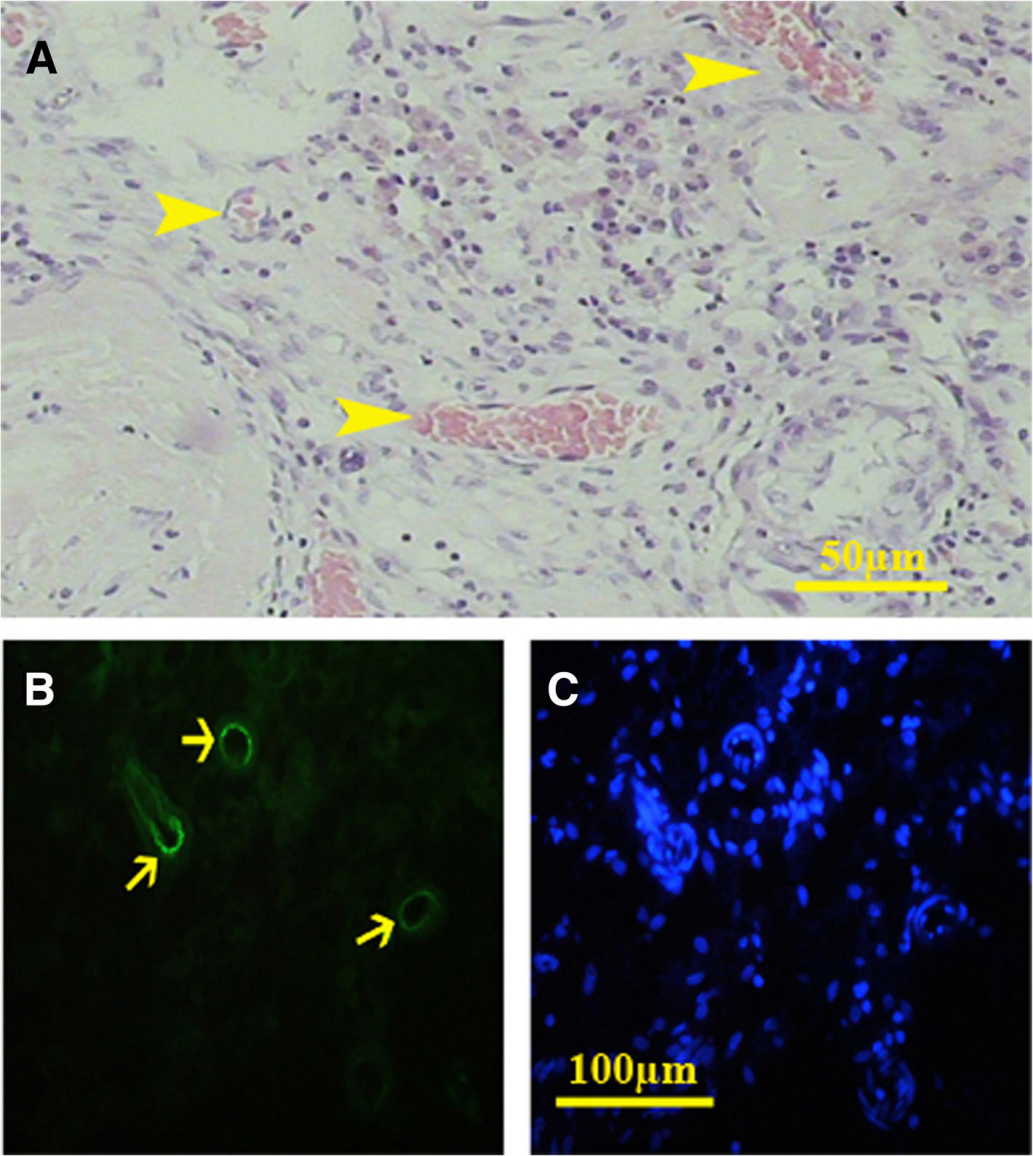
In vivo study showed that angiogenesis happened properly and different sections of the blood vessels were present in the transplanted scaffolds (**a**). H&E staining showed that all types of vessels were present in the transplanted sections. UEA lectin, as a marker of endothelial cells, also confirmed the angiogenesis (**b**, FITC-conjugated UEA; **c**, Hoechst)

The implants were penetrated by the blood vessels properly. UEA-positive cells covered the lumen of the blood vessels, confirming the fact that the angiogenesis happened after transplantation (Fig. 9). Also, some infiltrated immune cells were observed within both cell-free and cell-loaded scaffolds that indicated a mild inflammation.

**Fig. 10:**
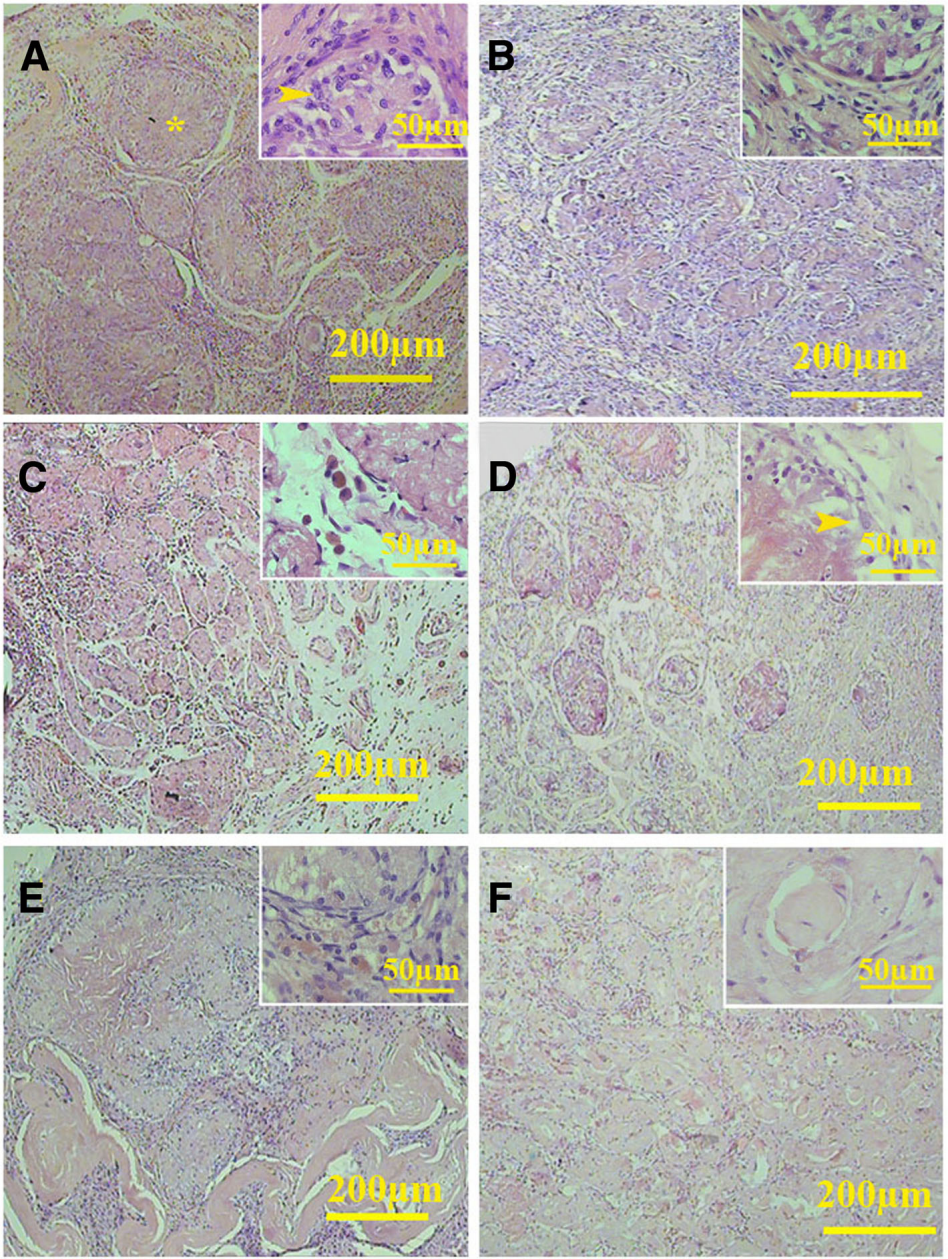
Histological sections of the cell-loaded (left series) and cell-free transplants (right series) on the liver. After 20 days, cells could be observed in the seminiferous tubules (*) of both cell-loaded (**a**) and cell-free (**b**) scaffolds. Some cells within the tubules had nuclei with close similarity to sertoli cells (Arrow). The cells surrounding the tubules had elongated nuclei similar to the myoid cells in the tunica propria. After 40 days (**c**, **d**), the sertoli-like cells had yet presented within the tubules. Between the tubules, some round eosinophil cells with round centrally located nuclei (arrow) could be also observed (**c**). At day 60 (**e**, **f**), some tubules occupied by fibrous tissue were present within the scaffolds (*)

**Fig. 11:**
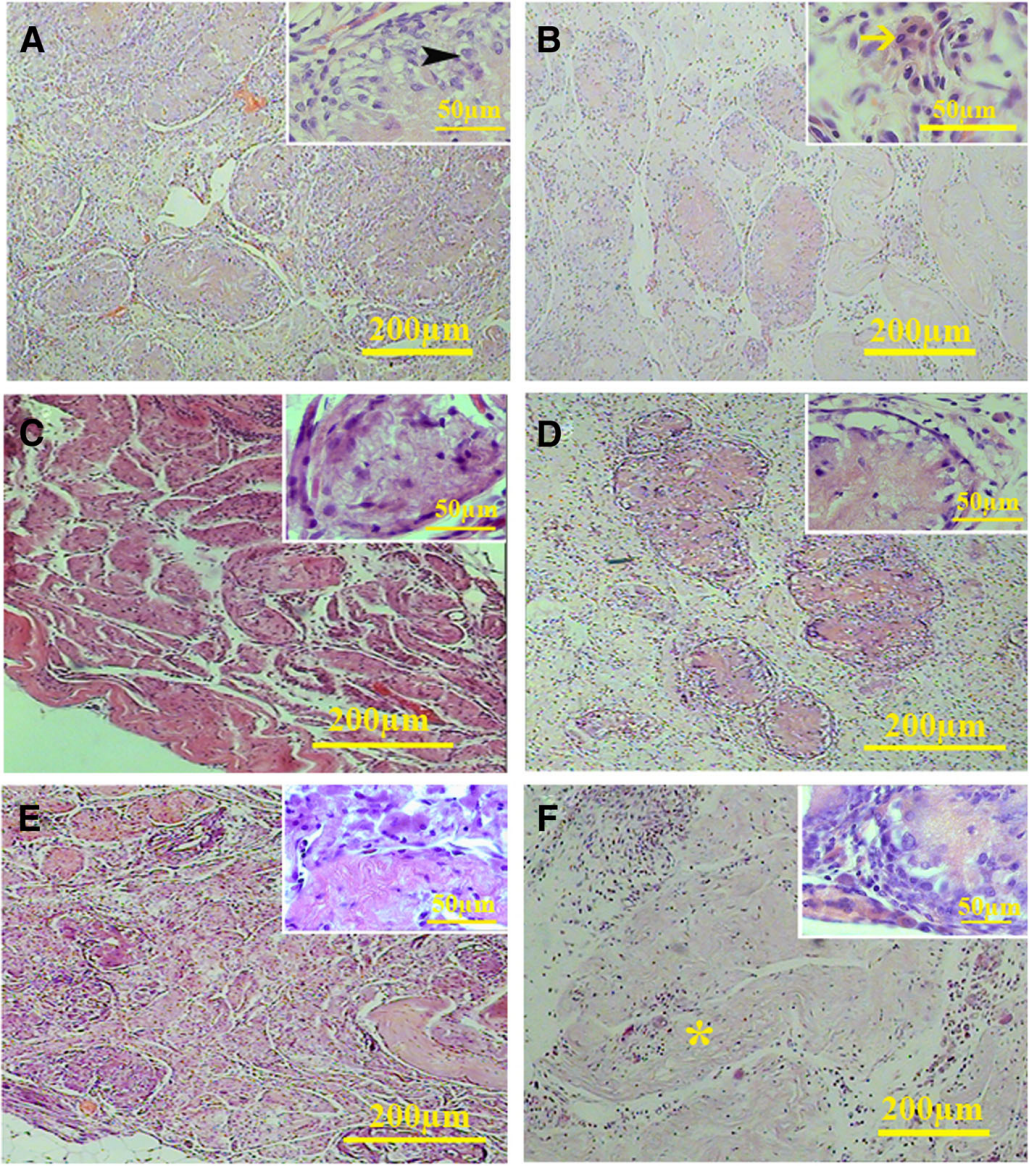
Histological sections of the cell-loaded (left series) and cell-free transplants (right series) on the mesentery. After 20 days (**a**, **b**), the cells within the seminiferous tubule showed sertoli-like cell phenotype (arrow head) along with round cells with leydig cell-like phenotype in the interstitium (arrow). A similar phenotype could be observed after 40 days (**c**, **d**). After 60 days, some tubules contained sertoli-like cells that had yet occupied some tubules. The percentage of the tubules occupied by fibrous tissue increased significantly at day 60 (asterisk) (**e** and **f**)

Histological study showed that the cells in the tunica propria had elongated nuclei similar to the myoid cells. Within the interstitium, both fibroblast and the round acidophilic cells with centrically located nuclei can be found (Figs. 10 and 11). Some other seminiferous tubules were occupied by fibrous tissue containing the fibers and elongated dark nuclei that might have belonged to the fibrocytes (Fig. 11e, f). The percentage of the fibrosis tubules was increased significantly in all conditions as the time progressed (all *P* < 0.001). Although the seminiferous tubules in both cell-loaded and cell-free implants contained the cells with sertoli cell phenotype, the percentage of tubules with fibrosis tissue was statistically the same in both conditions (transplanted on liver and mesentery) (Table 1).

**Table 1:** The percentage of the seminiferous tubules occupied by fibrous tissue in the various days

Days		20 days %	40 days %	60 days %
Liver	Cell-free scaffolds	29.24 ± 4.16*	67.30 ± 8.84	81.71 ± 1.66
	Cell-loaded scaffolds	30.97 ± .2.87*	58.75 ± 7.66	72.98 ± 7.19
Mesentery	Cell-free scaffolds	37.65 ± 14.1*	60.97 ± 5.27	89.15 ± 10.71
	Cell-loaded scaffolds	26.66 ± 4.91*	52.14 ± 22	67.69 ± 3.33

*Significant difference with the corresponding grafts after 60 days (*p* < 0.05)

**Fig. 12:**
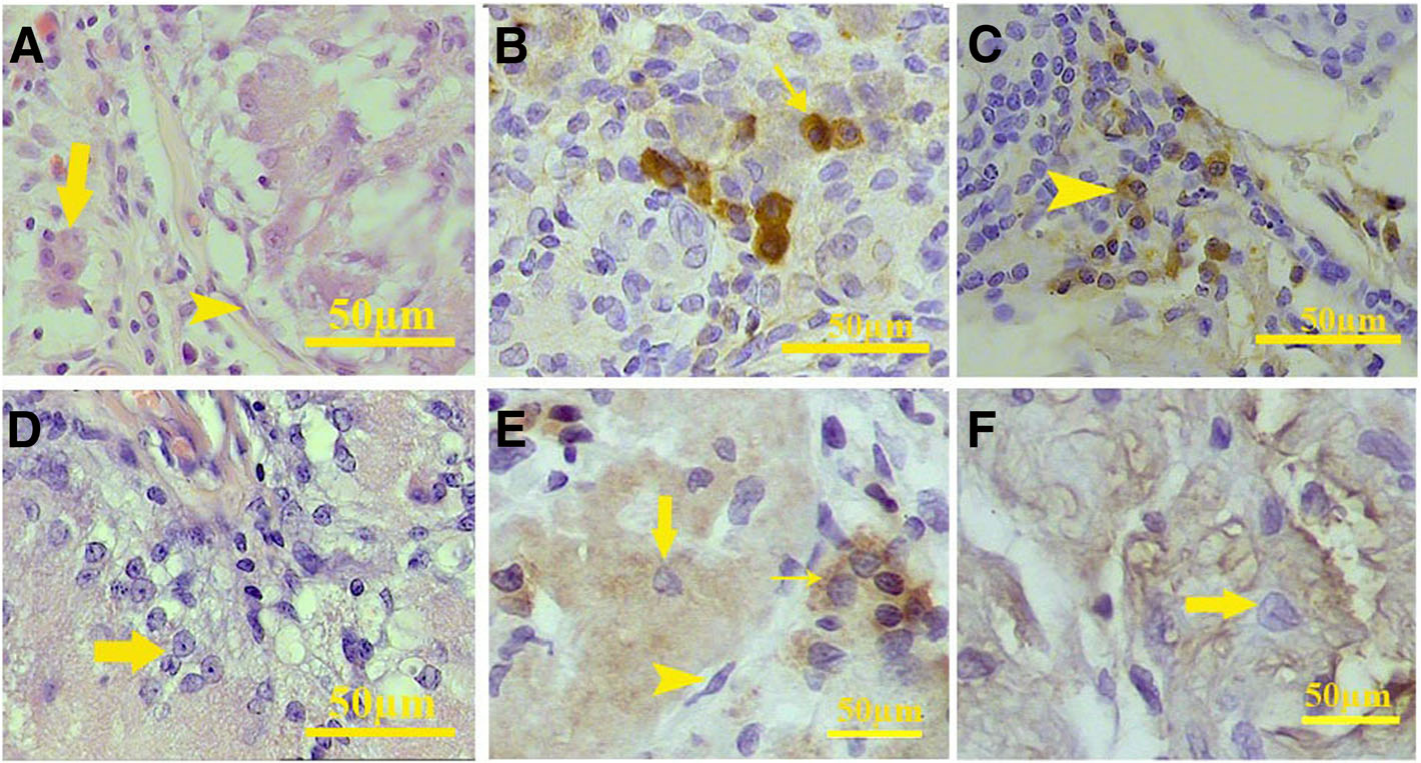
Higher magnification of the H&E staining showed that some cells with eosinophilic cytoplasm and round nuclei were present in the interstitium (arrow) after 20 days. Also, some elongated cells with close similarity with myoid cells present in the tissue surrounded each seminiferous tubule (arrowhead, **a**). Immunohistochemistry showed that some cells within the interstitium reacted with anti-inhibin antibody (**b**). Immunohistochemistry for CD68 revealed that a subpopulation of the round cells located between the tubules were macrophage (arrowhead, **c**). Also, H&E staining shows the presence of the cells with pale trigonal or round nuclei and expanded cytoplasm in some tubules in all samples (arrow, **d**). These sertoli-like cells were present within the tubules and react with anti-inhibin antibody (large arrow). Leydig-like cells that express inhibin (small arrow) and myoid-like cells (arrowhead) also were present in the transplanted scaffold (**e**). After 60 days, some seminiferous tubules contain inhibin expressing sertoli-like cells (arrow, **f**)

The round acidophilic cells had the phenotype similar to either leydig cell or macrophages (Fig. 12a). To distinguish these two types of cells, we performed immunostaining for inhibin and CD68. Although some cells were inhibin-positive (Fig. 12b), the others expressed CD68 (Fig. 12c). Therefore, within the interstitium, both macrophages and leydig-like cells were present.

In some seminiferous tubules, the cells with large pale nuclei and prominent nucleoli, similar to sertoli cell nuclei (Fig. 12d), were found. Some of the cells within the seminiferous tubules were inhibin-positive that may indicate they differentiated to the sertoli-like cells (Fig. 12e, f). To find the possible differentiation of the cells to post-mitotic cell lineages, we stained the implant with PNA lectin that is the marker of the acrosome and acrosomal granules. None of the seminiferous tubules contained PNA-positive cells that indicated there was no post-mitotic cell differentiation even after following for 60 days.

In the grafts transplanted in both the liver and mesentery, the area occupied by seminiferous tubules was statistically the same in the matched time series and the cell loading before graft had no significant influence on this criterion. As the time progressed, seminiferous tubules were kept in the implants on the liver; however, those implanted in the mesentery showed a significant reduction after 60 days compared to that after 20 days in both cell-loaded or cell-free conditions (*P* = 0.006 and 0.011, respectively, Table 2).

**Table 2:** The area that occupied by the seminiferous tubules in the various days

Days		20 days mm^2^/1 mm^2^	40 days mm^2^/1 mm^2^	60 days mm^2^/1 mm^2^
Liver	Cell-free scaffolds	0.026917 ± .0091802	0.023292 ± .0027741	0.012915 ± .003992
	Cell-loaded scaffolds	.033776 ± .0045181	0.028616 ± .0141431	0.016742 ± .0046878
Mesentery	Cell-free scaffolds	0.039351 ± .0078787*	0.031658 ± .00983333	0.013582 ± .0089382
	Cell-loaded scaffolds	0.042226 ± .0201494*	0.030711 ± .0120754	0.018701 ± .0130810

*Significant difference with the corresponding grafts after 60 days (P < 0.05)

**Fig. 13:**
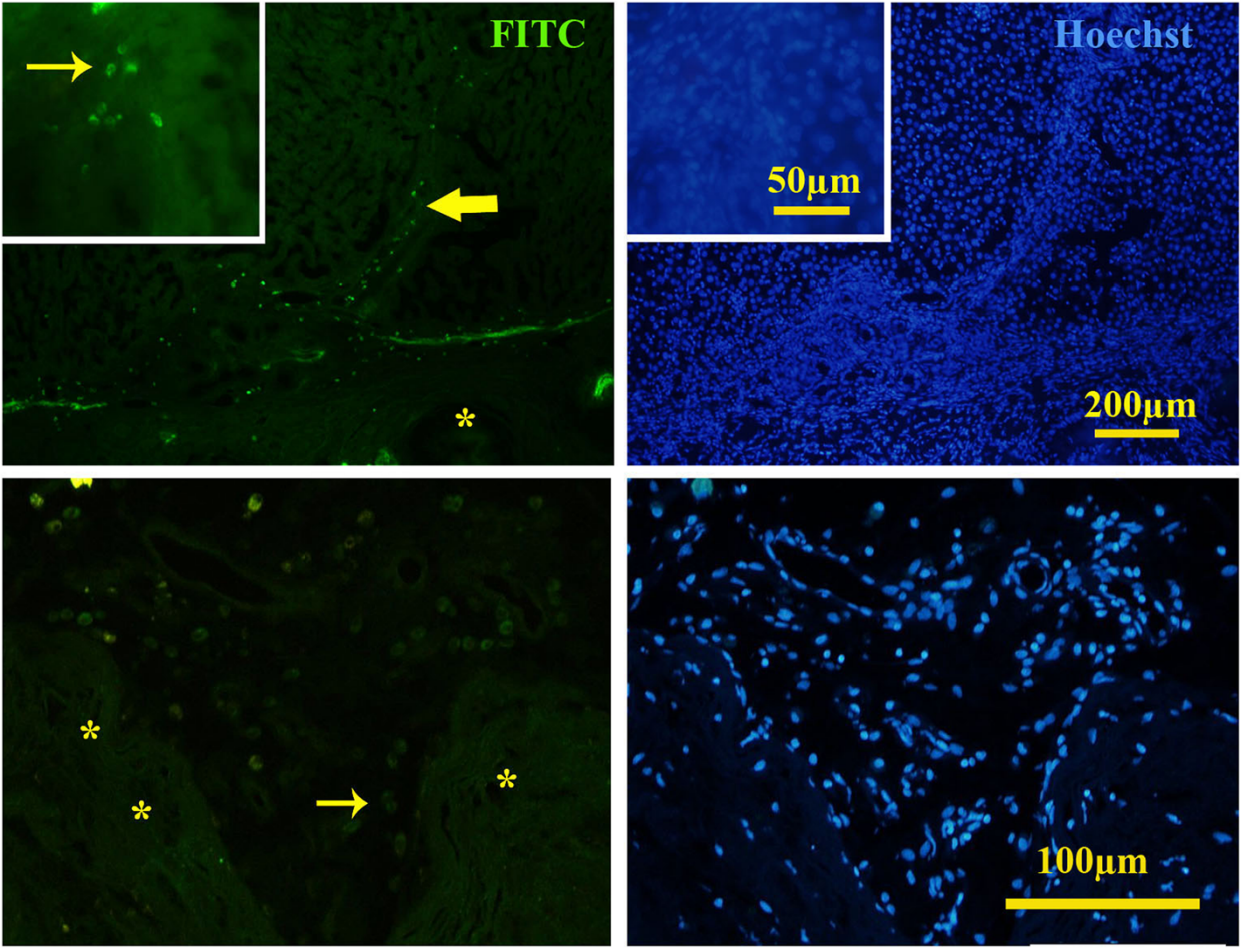
Immunofluorescence staining validation of the presence of DAZL-positive cells in the recellularized scaffold transplanted in the liver (above) on the day 20 and mesentery (bottom) on the day 60. Stream of DAZL-positive cells came from the edge of the liver toward the scaffold. The arrow shows the DAZL-positive cells, and the asterisk shows seminiferous tubules

**Fig. 14:**
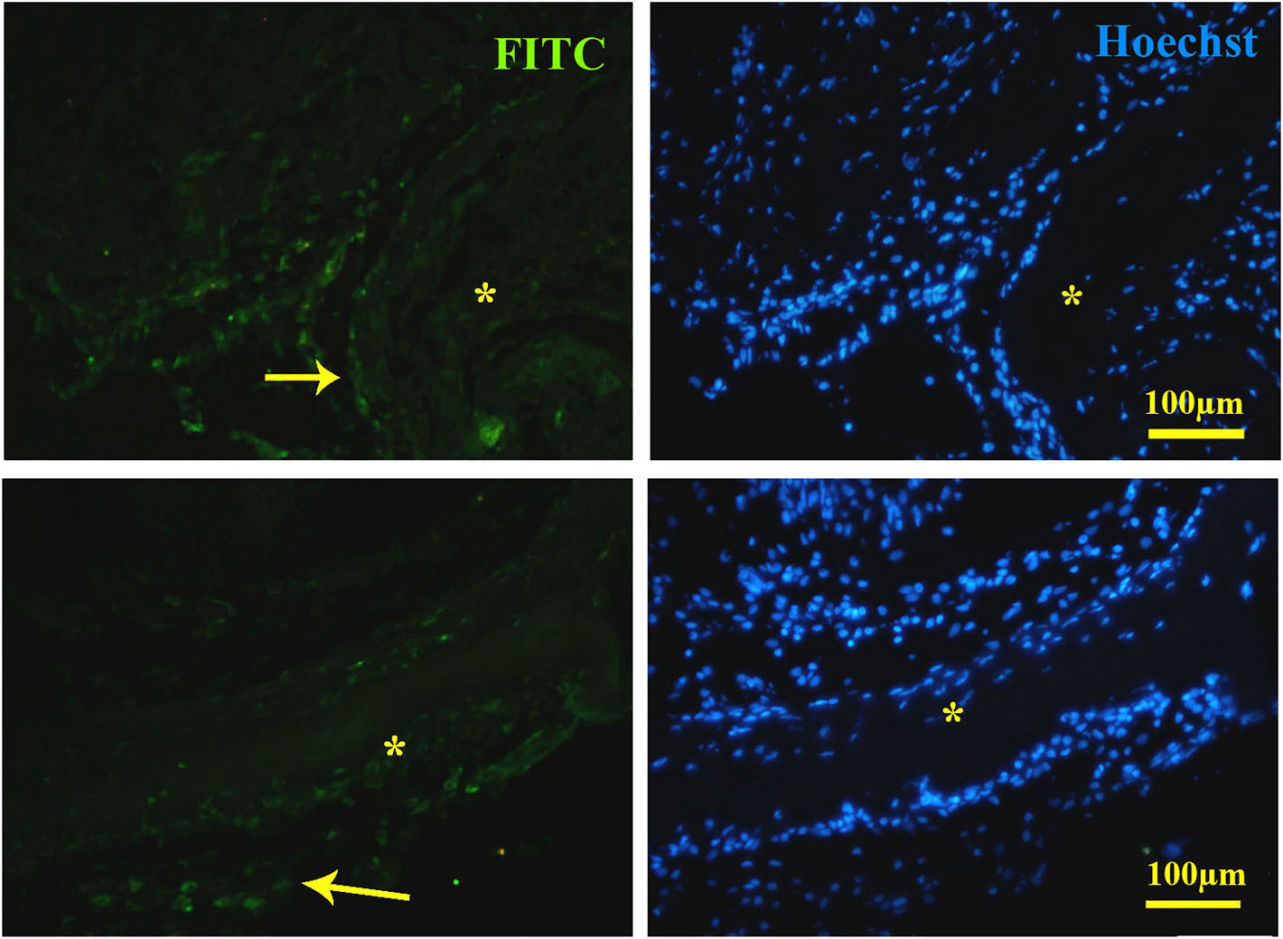
Immunohistochemistry showed that DAZL-positive cells migrated to the cell-free scaffolds that implanted into the liver (above) and mesentery (bottom). DAZL-positive cells were present in both the somniferous tubules and interstitium. Arrow shows the DAZL-positive cells, and the asterisk shows seminiferous tubules

Immunohistochemistry detected some migrating DAZL-positive cells toward the scaffolds in cell-loaded implants harvested from both mesentery and liver; however, after 60 days, the DAZL-positive cells disappeared in the scaffold transplanted on the liver (Fig. 13). DAZl-positive cells were also detected in both cell-free scaffolds transplanted in the both liver and mesentery after 40 and 60 days (Fig. 14).

## Discussion

Decellularized organs provide an appropriate ECM for tissue-specific cell differentiation. Detergents such as SDS have detrimental effects on the ECM constituents. In addition, trace amount of DNA and cellular antigens can cause serious side effects on the cell viability and functions [21]. The data from the current study indicated that in short term, the viability of the cells seeded on the decellularized scaffolds was less than those cultured in 2D conventional condition and it may be attributed to the presence of the cell debris and SDS trace within the scaffolds. However, as the time progressed, the cell viability increased in in vitro condition. In vivo tests also confirmed that the transplanted as well as host migrating cells could survive properly within the scaffolds. It has been shown that the extracellular matrix has chemical properties to trigger mitotic activity in the stem cells and migrating cells to the scaffold which is critical for tissue reconstruction [22]. Long-term culturing led to the removal of the trace of the SDS that improved the cell viability.

Our results indicated that the decellularized scaffolds remained when they were implanted in the liver and mesentery, but they were absorbed when they were transplanted in the subcapsular region of the kidney or in the subcutaneous part of the skin. The proper position for transplantation of the ectopic tissues such as engineered scaffolds is a critical point in the success of the cell-base or tissue replacement therapy. Although subcapsular space of the kidney has been suggested as an appropriate site for organ rudiment development [23] and subcutaneous part of the skin was used for ectopic transplantation of the cells and tissues in rodents [24], the best position for each ectopic tissue depends on the species and type of the tissue or transplanted cells [25]. Our data also showed that the partial reconstruction of the testis as well as angiogenesis successfully happened in the decellularized scaffolds transplanted in the liver and mesentery, as well.

This study reports a simple protocol for preparing a biocompatible matrix of the testes with minimal damage to the 3D structure and ECM constituents. In vitro assessment showed that the 2D monolayer cultures contained more viable cells compared with those cultured within 3D decellularized scaffolds on day 1, while on days 3 and 7, the number of viable cells was higher in 3D scaffolds. It seems that the cells reached confluency in 2D condition; therefore, there was no suitable space for the cell growth. The cells seeded on the 3D scaffolds had sufficient space for further growth. ECM is an essential factor for differentiation, cellular behavior, and gene regulation [26]. Also, another study pointed out that the maintenance of other compounds in ECM, such as vascular endothelial growth factor, could play an important role in the migration, reproduction, and differentiation of the cells grown during the in vivo post-transplantation process [27]. Here, the whole rat testis was perfused via non-ionic (Triton X100) and ionic (SDS) detergents as well as freeze and thaw and DNase to make the biomimetic scaffold. The freeze and thaw, as a physical process, lyses the cells and accelerates the penetration of the chemical solutions [21]. The use of DNase in the final stages of decellularization reduced the remaining of DNA fragments [21]. The effective removal of DNA and cell debris from the decellularized tissue led to better migration of the host cells to the scaffold and also reduced the inappropriate immune response [28, 29]; however, we also found a mild immune cell infiltration including CD68-positive macrophages and lymphocytes, as well. DNA content of less than 50 ng/mg dry weight was considered as an objective measurement for the effective elimination of cellular components [30]. Although there is no single optimized method for decellularization, we and others removed the cells effectively with minimal damage to the ECM [31–33]. A big challenge for optimizing the decellularization techniques is to find the balance between effective elimination of cellular components and maintenance of ECM materials.

We found that the 3D ultrastructure of the testis remained with minimal damage after decellularization which is consistent with previous studies [34, 35]. Spermatogenesis has been shown to happen in a 3D microenvironment that provides appropriate mechanical stress for the sperm development. Spermatogenesis was reported to be improved in 3D collagen type I hydrogel [36]. Besides, the basal lamina of the seminiferous tubules provides a niche for spermatogenesis [7]. Also, the functions of the testis-specific cells such as leydig cells were modified by culturing on the ECM constituents such as fibronectin, laminin, or collagen type IV [37]. Collagens also regulate the sertoli cell functions such as blood-testis barrier dynamic. Laminin has been suggested to have a role in spermatogenesis and germ cell perturbation [38]. Importantly, the isolated mouse neonatal testicular cells were reported to be reconstructed within 3D collagen matrix [39]. Laminin, fibronectin, and GAGs act as adhesion molecules that have been reported to promote the cell attachment and migration [40]. Both in vitro and in vivo experiments revealed that the morphology of the bone marrow-derived MSCs cultured within the decellularized scaffolds was regulated by the position of the cells within the scaffolds. The cells in the seminiferous tubules had spherical phenotype, and those in the interstitium or tunica propria had fibroblast-like morphology that may be attributed to the cell interaction with ECM. Also, some cells with the phenotype of leydig cells and the capability to express inhibin were found in the interstitium. It has been shown that the rat leydig cells express inhibin [41]. Sertoli-like cells within the seminiferous tubules were also detected that expressed inhibin, as well. It seems that the ECM constituents of the scaffolds have the ability to induce testis-specific cell differentiation. However, beside the role in the hypothalamic–pituitary–gonadal axis regulation, inhibin has been demonstrated to involve in immune cell differentiation and coordinate the tolerance versus immunity [42, 43]. Therefore, the presence of the inhibin-positive cells in the graft may be more related to their immunomodulatory role rather than leydig or sertoli cell differentiation.

The other constituents of the decellularized testis were GAGs that were preserved with minimal damage. GAGs have been shown to surround the sertoli and leydig cells in developing rat gonad [44], indicating the importance of such a component in testis-specific cell differentiation. GAGs can influence spermatogenesis by sequestrating the growth factors and protecting them from protease digestion [44]. Researchers have believed that GAG not only affects the cell adhesion, proliferation, and differentiation, but also plays a crucial role in the cell fate [45].

A line of evidence showed the presence of extra gonadal germ cell population in the bone marrow and peripheral blood. In female mouse, this cell population has been reported to be influenced by the estrous cycle, can migrate to the ovary, and is involved in the oocyte differentiation [46]. DAZL-positive cells have detected human decellularized ovarian scaffold transplanted in the ovariectomized rat model [47]. These cells have been also detected in the adult male mouse [48] and human male fetus bone marrow [49]. Besides, a pluripotent cell population, called very small embryonic-like stem cells (VSEL), was isolated from the peripheral blood and bone marrow, which has a tendency to differentiate into the germ cells [50]. This cell population expresses DAZL and can migrate under stress or damaged conditions [51]. Our data revealed a stream of DAZL-positive cells migrating from the liver or mesentery toward the decellularized scaffold, and it is possible that they are derived from VSEL or DAZL-positive cells circulating in the blood.

Promising results of good ECM preservation and successful in vitro recellularization encouraged us to test in vivo transplantation of the testis scaffold. Qualitative observation showed that following transplantation of the cell-loaded and cell-free scaffolds to the liver and mesentery, the infiltration of immune cells (mainly macrophages, epithelioid cells and lymphocytes) was low. Moreover, the researchers suggested that the decellularized scaffolds have anti-inflammatory and immune-repressive effects [52]. The underlying mechanism may be the reduction of MHC I and II molecules after decellularization [53, 54].

The data of the current study showed that the MSCs could not differentiate into post-mitotically cell lineages. MSCs from various sources were injected to the testis of different infertile animal models. In most of the cases, various combinations of growth factors were used to induce germ cell differentiation before or during the cell injection [55]. Besides, co-culturing systems with other cell types, such as sertoli cells, were previously performed to induce trans-differentiation of MSCs into germ cells [56], and then, the differentiated cells were transplanted into the busulfan-induced azoospermic rats [57]. Yet, some other studies have revealed that the injected undifferentiated MSCs into the busulfan-induced rats improved the spermatogenesis [58]. This may be due to the effects of cytokines that MSCs produced within the host testis. However, the differentiation of the MSCs into the sperms was not confirmed [58]. In this study, we did not use any growth factor supplementation or co-culture system and we found that DAZL-positive cells migrated from the host to both cell-loaded or cell-free decellularized testis. It may be helpful to suggest that using enriched decellularized scaffolds with growth factors may promote the DAZL-positive cells toward post-mitotic cell lineages.

## Conclusion

Data from the current study showed that the cell component was eliminated in the rat testis, while the three-dimensional structure and constituents of the ECM remained with minimal damage after decellularization. Since the scaffold could be recellularized successfully and it exhibited good cytocompatibility, the in vivo tests showed some testis-specific cells such as inhibin-positive cells within the scaffolds. Besides, the scaffold provided a microenvironment for DAZL-positive cell migration; however, we did not find any post-mitotic spermatogenic cell lineage. The scaffold may provide a valuable boon for in vitro and in vivo spermatogenesis. Our outcomes can promote the understanding of the role of ECM in testicular function, as well.

## Abbreviations

ART: Assisted reproductive techniques
DAB: Diaminobenzidine
DAZl: Deleted in Azoospermia-Like
DDW: Deionized distilled water
DMEM: Dulbecco’s modified Eagle’s medium
DNase: Deoxyribonuclease
ECM: Extracellular matrix
FBS: Fetal bovine serum
GAGs: Glaycosaminoglycans
H&E: Hematoxylin and Eosin
MSCs: Mesenchymal stem cells
MTT: 3-(4, 5-Dimethylthiazolyl-2)-2, 5-diphenyltetrazolium bromide
OCT: Optimal cutting temperature
OD: Optical density
PAS: Periodic acid schiff
PBS: Phosphate-buffered saline
PNA: Peanut agglutinin
SDS: Sodium dodecyl sulfate
SEM: Scanning electron microscopy
UEA: *Ulex europaeus* agglutinin
VSEL: Very small embryonic-like stem cells
